# The Role of Nucleases and Nucleic Acid Editing Enzymes in the Regulation of Self-Nucleic Acid Sensing

**DOI:** 10.3389/fimmu.2021.629922

**Published:** 2021-02-26

**Authors:** Pauline Santa, Anne Garreau, Lee Serpas, Amandine Ferriere, Patrick Blanco, Chetna Soni, Vanja Sisirak

**Affiliations:** ^1^CNRS-UMR 5164, ImmunoConcEpT, Bordeaux University, Bordeaux, France; ^2^Department of Pathology, New York University Grossman School of Medicine, New York, NY, United States; ^3^Immunology and Immunogenetic Department, Bordeaux University Hospital, Bordeaux, France

**Keywords:** DNases, RNases, systemic lupus erythematosus, DNA sensing, RNA sensing, interferonopathies, aicardi goutieres syndrome, toll-like receptors

## Abstract

Detection of microbial nucleic acids by the innate immune system is mediated by numerous intracellular nucleic acids sensors. Upon the detection of nucleic acids these sensors induce the production of inflammatory cytokines, and thus play a crucial role in the activation of anti-microbial immunity. In addition to microbial genetic material, nucleic acid sensors can also recognize self-nucleic acids exposed extracellularly during turn-over of cells, inefficient efferocytosis, or intracellularly upon mislocalization. Safeguard mechanisms have evolved to dispose of such self-nucleic acids to impede the development of autoinflammatory and autoimmune responses. These safeguard mechanisms involve nucleases that are either specific to DNA (DNases) or RNA (RNases) as well as nucleic acid editing enzymes, whose biochemical properties, expression profiles, functions and mechanisms of action will be detailed in this review. Fully elucidating the role of these enzymes in degrading and/or processing of self-nucleic acids to thwart their immunostimulatory potential is of utmost importance to develop novel therapeutic strategies for patients affected by inflammatory and autoimmune diseases.

## Introduction

The innate immune system is the first line of defense of an organism against microbial infections. Upon the sensing of microbial components called pathogen associated molecular patterns (PAMPs) by pattern recognition receptors (PRR), the innate immune system produces inflammatory mediators viz. type-I Interferons (IFN-I) that are critical for the activation of antimicrobial immunity. Multiple PRR have evolved to recognize microbial nucleic acids (NAs) which represent a major PAMP (1). This ability of PRR to detect microbial genetic material confers a great advantage to the host, by enabling the activation of the immune system against a broad range of microbes. However, this specificity of PRR comes with an imminent risk, as PRR specialized in NA sensing do not robustly discriminate between self (endogenous) and foreign NAs (2). To avoid the aberrant immune activation by self-DNA, DNA sensors are strategically located in cellular compartments commonly devoid of self-DNA such as the cytosol and endolysosomes (3). Furthermore, NA sensors preferentially recognize sequences and/or structures that are enriched in microbial genetic material including un-methylated CpG motifs (abundant in microbial DNAs) and uncapped 5′ tri- and bi-phosphates (abundant in viral RNAs) (2). It was recently reported that histones also prevent aberrant activation of inflammatory responses by genomic DNA (gDNA), through the inhibition of intracellular DNA sensors (4–7). While these mechanisms clearly prevent abnormal activation of NA sensors by endogenous NAs, they are not sufficient given the abundance and availability of self-NAs. Indeed, the natural turnover of cells accounts for millions of dying cells every day that release significant amounts of their genetic material, while cellular stress conditions including genotoxic and oxidative stress, autophagy, etc. can lead to exposure of nuclear and mitochondrial genetic material into the cytosol. Therefore, the relative abundance and antigenicity of self-NAs must also be tightly regulated to limit their immunostimulatory potential and prevent the development of inflammatory and autoimmune disorders (3). This role is ensured by nucleases (DNases and RNases) and NA-editing enzymes, that function extra and intracellularly to prevent self-NA-mediated autoimmunity. After briefly describing the NA sensors and the main sources of potentially immunostimulatory self-NAs, this review will focus on the nucleases and NA-editing enzymes involved in the regulation of self-NA immunogenicity. Particularly we will describe their expression profiles, biochemical properties, functions and mechanisms of action. Moreover, we will simultaneously address how dysregulation and deficiencies in these enzymes contribute to inflammatory and autoimmune diseases. The functional analysis of nucleases' and NA-editing enzymes will be further extended to cancer, another pathological context that involves NA sensing. Finally, the potential therapeutic avenues to overcome pathologies mediated by nucleases and NA-editing enzyme dysfunction will be discussed.

## Innate Immune Sensors of Nucleic Acids

There are two major subtypes of NA sensing PRR that were classified according to their subcellular localization, including cytosolic and endolysosomal NA sensors. Their function, regulation and signaling pathways were recently thoroughly reviewed (2), therefore we will briefly describe them to understand the function of nucleases and NA-editing enzymes in the regulation of NA sensing.

***Cytosolic NA sensors*** are widely expressed across immune and non-immune cells, and recognize cytosolic NAs. Cytosolic double stranded (ds)RNA is sensed by RIG-I-like receptors (RLRs) including RIG-I (retinoic acid-inducible gene I) and MDA5 (melanoma differentiation-associated protein 5). RIG-I is activated by 5′ tri-phosphorylated, 5′ di-phosphorylated and to lesser extent by 5′-OH short dsRNA (8), while MDA5 recognizes highly branched forms of dsRNA of >1 kbp (9), none of which are found endogenously. RIG-I and MDA5 interact with the mitochondrial antiviral signaling protein (MAVS) on the outer mitochondrial membrane, which activates the MAVS signaling complex leading to IFN-I and proinflammatory cytokine production (3). The principal cytosolic DNA sensor is cGAS (cyclic GMP-AMP synthase), which upon DNA recognition, synthesizes cyclic GMP–AMP (cGAMP), that functions as a second messenger to activate the stimulator of IFN genes (STING) on the endoplasmic reticulum (ER). STING engagement also causes IFN-I production (10). Another cytosolic dsDNA sensor is absent in melanoma 2 (AIM2), which upon activation engages the inflammasome to cause production of interleukin (IL)-1β, IL-18 and other inflammatory cytokines (11). However, all mouse AIM2-like receptors (ALRs) and human IFI16 are dispensable for the IFN-I response to intracellular DNA (12). Most cytosolic RNA sensors mediate responses to various classes of RNA viruses, whereas cytosolic DNA sensors induce antiviral immunity against DNA viruses and retroviruses. Recent reports show that cGAS can also be localized to the nucleus preferentially to centromeric DNA and LINE-DNA repeats upon disruption of nuclear membrane during cell migration and interphase of the cell cycle. Nuclear localization of cGAS has been suggested as a process that might regulate tonic or basal IFN-I signaling (13–15).

***Endolysosomal NA sensors*** consist of the Toll-like receptor (TLR) family members TLR3, −7, −8, −9, and −13. They are mainly expressed by immune cells and identify endocytosed NAs. TLR9 preferentially recognizes unmethylated CpG dinucleotides in dsDNA sequences (16, 17), while TLR3 is activated by 39-48bp of dsRNA (18). TLR8, which is reportedly non-responsive to stimulation by RNA in mice contrary to humans, is expressed in human monocytes, dendritic cells (DCs) and neutrophils, whereas TLR7 is widely expressed in immune cells of both humans and mice (19). Both TLR7 and TLR8 recognize single stranded (ss)RNA and its degradation products. Recent biophysical and biochemical studies have identified critical differences between the two, wherein TLR8 recognizes ssRNA uridine and short oligonucleotides (20), while TLR7 is preferentially activated by guanosine and its derivatives (21). TLR13 is a murine-specific endosomal sensor of bacterial 23S rRNA (22). Ligand binding to TLR7, −8, −9, and 13 initiates signaling via the adaptor protein MyD88 (myeloid differentiation primary response protein 88), while TLR3 signals via the adaptor protein TRIF (TIR domain-containing adaptor inducing interferon-β). Both TRIF and MyD88 pathways lead to NF-κB-mediated inflammatory cytokine production and IFN regulatory factor (IRF)-3/7-mediated IFN-I production, which are both necessary for antimicrobial immune responses (23–25).

## Sources, Forms and Immunogenicity of Self Nucleic Acids

Most NA sensing PRR do not properly discriminate between microbial and endogenous NAs. Accordingly, endogenous NAs were reported to activate NA sensing pathways and contribute to inflammatory and autoimmune syndromes (2). In this section we will discuss the main sources, forms and properties of endogenous NAs and how they may gain access to cellular compartments containing NA sensors.

***Cell free extracellular NAs*** were first identified in the circulation of a patient with leukemia in 1931 by Labbe et al. (26) and several years later Mandel and Metais were able to extract both DNA and RNA from the plasma of healthy patients (27). These pioneering studies indicated that endogenous NAs are present systemically and that their quantities are altered in pathological settings. Since then, technological advances in purification, quantification and sequencing has led to better characterization of circulating cell free (cf)-NAs (28).

*CfDNA* is relatively abundant in the circulation of healthy individuals, ranging from 5 to 10 ng/mL of plasma (29). Studies using gel electrophoresis (30–32) and DNA sequencing (33–35) have indicated a laddering pattern of cfDNA reminiscent of apoptotic DNA products with a dominant DNA species of 167bp corresponding to the length of DNA associated with a single chromatosome. Further examination of cfDNA in sex-mismatched bone marrow (BM) recipients (36) and its methylation profiles (37, 38), have revealed that 80% of cfDNA originates from dying hematopoietic cells, including granulocytes, and lymphocytes. Importantly, these contributions can shift during pregnancy and aging and in pathological contexts (cancer, transplantation, and autoimmune syndromes) (39). In addition, to gDNA, mitochondrial DNA (mtDNA) is readily detected in the circulation of healthy individuals (40), originates from dying cells (41) and was found to be 56-fold more abundant than circulating gDNA (42).

Various forms of gDNA and mtDNA are present in the circulation. They can be free (“naked”), associated with proteins such as histones and HMGB1 (High mobility group box 1) for gDNA and TFAM (Mitochondrial transcription factor A) for mtDNA, and finally they may be associated with microparticles (MPs). MPs are extracellular vesicles typically between 0.1 and 1.0 μm in diameter that originate from the outward budding of the plasma membrane (43). DNA isolated from MPs shows a laddering pattern (31), suggesting that MP-associated DNA is derived, in part, from apoptotic cells. DNA that is associated with MPs can be exposed on their surface and/or remain inside, and usually consists of chromatin fragments containing histones and DNA associated proteins (HMGB-1 and TFAM) (44–46).

While cfDNA derived from apoptotic cells arises naturally, infection, inflammatory conditions, and cancer may lead to release of circulating DNA with different properties. It was reported that necrosis (accidental cell death characterized by a rapid loss of plasma membrane integrity in the absence of nuclear fragmentation) of cancer cells contributes to the accumulation of larger fragments (>1 kb) of cfDNA in the circulation (47). Furthermore, neutrophils undergo a specific cell death process called NETosis, which results in the release of DNA in the form of neutrophil extracellular traps (NETs) (48). These structures facilitate trapping of bacteria, and thus are important for antimicrobial immunity (48). In addition to gDNA, NETs are composed of mtDNA, and both can be associated with the anti-microbial peptide LL37 (48) which protects such DNA from degradation (49). In addition, DNA that is extruded by neutrophils is oxidized, further facilitating its protection from nucleases (50) and enhancing its immunostimulatory potential (51).

Naked forms of gDNA found in the circulation are mostly inert and display relatively low immunostimulatory capacities (52). On the other hand, mtDNA which shares many features with bacterial DNA, harbors elevated levels of unmethylated immunostimulatory CpG motifs (53), and was reported to activate TLR9 (54). The association of cfDNA with HMGB1 and its mitochondrial counterpart TFAM also contributes to the immunogenicity of cfDNA. Indeed, HMGB1 and TFAM were shown to promote cfDNA transport into intracellular compartments and to enhance TLR9 activation by self-DNA particularly in plasmacytoid dendritic cells (pDCs) which are specialized in IFN-I production (55, 56). Moreover, they confer self-DNA-specific secondary structures capable of activating cGAS (57). DNA associated with MPs also carries the potential to activate innate immune responses mainly through TLR9 (58). This ability is likely mediated by the endocytosis of MPs, which grants access to the endolysosomal compartment for further processing but does not facilitate the stimulation of cGAS and other cytosolic DNA sensors. In addition to their forms, it is becoming clear that the size of cfDNA directly impacts its immunogenic potential. Longer DNA fragments stimulate cGAS mediated IFN-I production more efficiently (59) and bind with higher affinity to dsDNA autoantibodies that accumulate in autoimmune syndromes such as systemic lupus erythematosus (SLE) (60). Finally, DNA that is extruded during the process of NETosis is also highly immunogenic and induces IFN-I production in a TLR9- (61, 62) and cGAS-dependent manner (51).

*CfRNA* levels, sources, physical forms and its potential to become immunostimulatory is less characterized compared to cfDNA. Many attributes of RNA make its presence in circulation unlikely. RNA is a short-lived highly labile molecule susceptible to alkaline pH, heavy metal ions, and RNases abundant in circulation (63). In spite of its instability, several studies have identified numerous RNA species in the plasma, the most prevalent of which include miRNA and piwiRNA and to a lesser extent mRNA, lncRNA, rRNA, and tRNA (64). A study from the Lo group found that “naked” RNA was degraded in human plasma after only 15 s of incubation, suggesting that any RNA normally found in plasma must be protected (65). Indeed, after passing plasma through a 0.2 μm filter there was a 10-fold reduction in the amount of RNA recovered, suggesting a mix of MP-associated and MP-free RNA in circulation (65). Several studies have also shown that cell-free miRNA is particularly resistant to nucleases (66), due to its association to proteins rather than with vesicles, indicating multiple methods of cfRNA stabilization may exist. The immunostimulatory capacity of circulatory MP-associated RNA has been investigated to some degree in the context of RNA specific TLR activation; however this as well as the role of MP-free circulating RNA species remain active areas of research (67, 68).

***Intracellular NAs*** represent a much larger pool of self-NAs residing within cells in the form of gDNA, mtDNA and RNA. gDNA and mtDNA are segregated from intracellular DNA sensors and post-transcriptional modifications of endogenous RNA restrict its capacity to stimulate cytosolic RNA sensors (2). However, gDNA and mtDNA are known to gain access to the cytosol. Micronuclei are small organelles surrounded by a nuclear envelope containing condensed nuclear DNA, and “speckles” are less condensed cytosolic DNA structures (69), which form during mitosis and DNA damage, respectively. Exposure of gDNA in cytosolic “speckles” and/or micronuclei upon rupture of the nuclear envelope (69) stimulate cGAS and induce IFN-I production (70–72). Cell stress and cell death (54) also participate in the release of mtDNA into the cytosol. The proximity of mtDNA to reactive oxygen species (ROS) makes it more susceptible to oxidation, a modification that makes mtDNA more resistant to degradation by nucleases (50), and boosts its immunostimulatory potential. Accordingly, once in the cytosol, unmodified mtDNA stimulates the cGAS-STING pathway (73, 74), while its oxidized counterpart acquires the capacity to activate the NLR family pyrin domain containing 3 (NLRP3) inflammasome (75). As discussed previously mtDNA is rich in un-methylated CpG motifs that constitute the main ligand for TLR9. In addition to the uptake of extracellular mtDNA into endolysosomal compartments containing TLR9 (58), mtDNA can be directly transported to endolysosomes by autophagy and trigger TLR9 activation if mtDNA degradation during this process is incomplete (54).

Endogenous RNAs, as discussed previously, are poor stimulators of intracellular RNA sensors due to their biochemical properties. Nevertheless, non-coding retroelements that make up a large portion of the human genome, are known to produce dsRNA duplexes resembling viral dsRNA. Although not all retroelements are active in this way, the dsRNA products of many have been shown to activate intracellular NA sensors (76). There is some evidence that retroelement-derived dsRNA duplexes, if not degraded, are recognized by intracellular RLRs (77, 78) and trigger inflammatory cytokines and IFN-I production. Recently, mtdsRNA was also shown to signal through MDA5-MAVS to induce IFN-I production if not degraded by mitochondrial RNA-degradosome machinery (79). Finally, endogenous retroelements not only form dsRNA product but also contribute to the generation of complementary DNA (cDNA) upon reverse transcription. These cDNA were also described to be an important source of intracellular DNA, the levels of which if not properly regulated may contribute to the aberrant activation of the cGAS-STING pathway (80, 81).

**Figure 1:**
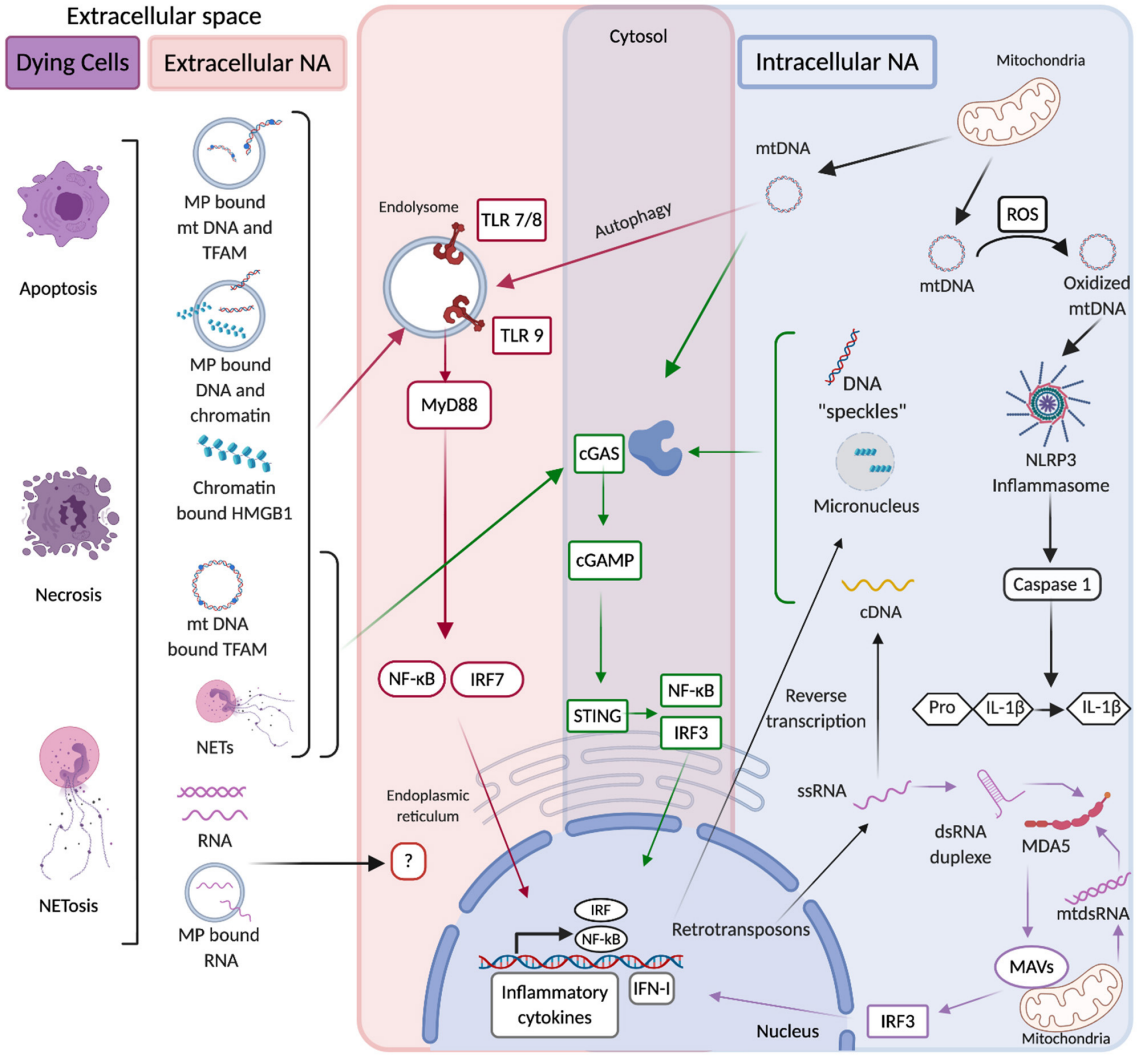
In red are extracellular source of NAs that originate from dying cells in multiple forms including free, microparticle (MP)-associated, Neutrophil Extracellular Trap (NET)-associated, and protein-associated (Histones, HMGB1, and TFAM). Such NAs can be internalized into endolysosomes where they are recognized by Toll like receptors (TLR), which via MyD88 activate NF-kB and IRF7 transcription factors that upon their translocation to the nucleus induce the production of inflammatory cytokines and type I interferons (IFN-I). The detection of extracellular RNA by NA-sensing PRRs is poorly documented. In blue are represented intracellular sources of NAs. They originate from mitochondria and such mtDNA in the presence of ROS (reactive oxygen species) can be oxidized and acquire the ability to activate NLRP3 inflammasome, which triggers caspase 1-mediated cleavage of pro-IL-1β into active IL-1β. mtDNA can also activate cGAS which upon detection of DNA produces cGAMP that is specifically recognized by STING. STING stimulation triggers IRF3-mediated IFN-I production and NF-kB-mediated inflammatory cytokine production. Upon autophagy mtDNA can gain access to endolysosomal compartments and stimulate TLR9 as well. Furthermore mitochondrial double-stranded RNA (mtdsRNA) if not degraded by mitochondrial RNA-degradosome machinery can activate the RNA sensor MDA5 which via the adaptor molecule MAVS activates IRF3-mediated IFN-I production. Endogenous LTR-retrotransposons are also a source of intracellular NAs, they can lead to the production of dsRNA duplexes that activate IFN-I production after their sensing by MDA5. In purple are common pathways of intracellular and extracellular NA sensing. mtDNA and NET-associated DNA from the extracellular space are internalized in the cytosol and activate cGAS. The same pathway can be activated by ssDNA originating from the reverse transcription of endogenous LTR-retrotransposons and by nuclear DNA released into the cytosol during stress conditions in forms of micronuclei or “speckles”.

There are thus multiple sources of endogenous NAs that can be distributed both extra and intra-cellularly (Figure 1). They assume various forms that regulate their half-life, distribution and immunostimulatory properties. Endogenous NAs are readily detected extracellularly, but in pathological contexts their abundance is commonly increased, and their physical form is altered. In addition, impairment of the intracellular distribution of NAs, renders them accessible to intracellular NA sensors. According to their source, form, distribution and modifications, endogenous NAs exhibit differential immunostimulatory properties. Nevertheless, most endogenous NAs are capable of activating innate immune receptors and stimulate the secretion of inflammatory cytokines (Figure 1). Due to the imminent danger such NAs pose to the host, their availability and immunogenicity must be subjected to stringent regulation.

## Nucleases: Safety Nets That Prevent Self Nucleic Acid Immunogenicity

There are multiple nucleases and NA-editing enzymes that regulate the abundance and the immunostimulatory potential of self-NAs. They can be subdivided in two classes, extracellular and intracellular NA processing enzymes. Their expression profiles, functions and their contribution to pathologies in both mice and humans are summarized in Table 1.

**Table 1:** Comparative analysis of murine and human nucleases and NA-processing enzymes.

**Nuclease**	**Main tissue expression (mice and humans)**	**Main cell types (mice and humans)**	**Substrates**	**Mutations in humans and associated pathologies**	**Deficiency in mice and associated pathologies**	**Signaling pathway activated upon deficiency**
DNASE1	Salivary glands; Kidney; Gut; Plasma	Exocrine cells; Paneth cells	Apoptotic cell-derived DNA; Neutrophil extracellular trap (NET) DNA	Systemic Lupus Erythematosus (SLE)^a^ (82)	SLE^a^ (83)	Unknown
DNASE1L1	Skeletal and Cardiac muscle	Myocytes	Liposome associated DNA (natural substrate unknown)	Pompe's disease^a^; ANCA-associated vasculitis (84–86)	Muscle weakness and reduced total body mass (87)	Unknown
DNASE1L3	Spleen; Liver; Plasma	Dendritic cells, Macrophages	“Naked” DNA; Nucleosome-bound form; Microparticle (MP)-associated DNA; NET-DNA	Pediatric-onset SLE; Systemic sclerosis (SSc), Rheumatoid Arthritis (RA) (88–99)	SLE (Strong serological features) (45, 100)	TLR7; TLR9; Myd88 dependent (45, 101)
RNASET2	Ubiquitous	Most cells	Extra and intracellular ssRNA	Cystic leukoencephalopathy (102)	Neuropathology and deficits in memory (103)^*^	Unknown
DNASE2A	Ubiquitous	Macrophages	Apoptotic cell-derived dsDNA and endogenous genomic dsDNA	Rheumatoid arthritis (RA); Anemia, Lupus nephritis (104–106)	Embryonic lethal; Anemia, Rheumatoid arthritis; Nucleic acid specific autoantibodies (107–111)	cGAS/STING, AIM2 and endolysosomal TLRs dependent (111–115)
DNASE2B	Lens	Lens fiber cells	Nuclear dsDNA	Unknown	Cataracts (116)	Unknown
PLD3/PLD4	Most tissue	Macrophages; DC	ssDNA originating from apoptotic cells	Rheumatoid arthritis (RA); Systemic sclerosis (SSc) (117–119)	Chronic immune activation resulting in inflammatory disease (120)	TLR9 dependent (120)
DNASE1L2	Epidermis	Keratinocytes	Nuclear dsDNA	Parakeratosis; Psoriasis. (121)	Hair and nail parakeratosis (122)	Unknown
TREX1	Ubiquitous	Ubiquitous	ssDNA derived from retrotranscribed retroelements	Aicardi Goutières Syndrome (AGS); Familial Chilblain Lupus (FCL), Retinal Vasculopathy with Cerebral Leukodystrophy (RVCL); SLE (123–130)	Lethal autoimmunity, Myocarditis, Lupus like disease; FCL-like disease (81, 128)	STING, cGAS, TBK1, IRF7, IRF3, and IFNAR1 dependent. (80, 81, 113, 128, 131, 132)
RNase H2 complex	Ubiquitous	Ubiquitous	RNA in RNA: DNA hybrid structures	Aicardi Goutières Syndrome (AGS), SLE (133, 134)	Embryonic lethal; IFN-mediated autoimmunity (135, 136)	cGAS/STING dependent (135, 136)
SAMHD1	Most tissue	Immune cells	dNTPs	Aicardi Goutières Syndrome (AGS); Familial Chilblain Lupus (FCL); Arthropathy (137, 138)	Spontaneous type-I IFN production and upregulation of ISGs; No inflammatory disease (139, 140)	Unknown
ADAR1	Ubiquitous	Ubiquitous	dsRNA duplexes	Aicardi Goutières Syndrome (AGS). (141)	Embryonic lethal (142)	MDA5; MAVS- dependent (143, 144)
RNA Exome	Ubiquitous	Ubiquitous	dsRNA	Trichohepatoenteric syndrome (145)	Unknown	RLRs (146)
Endo G	Ubiquitous	Ubiquitous	mtDNA	Unknown	Cardiac hypertrophy (147)	cGAS/STING (148)

*Blue are extracellular, Green are endolysosomal and yellow are intracellular nucleases or NA processing enzymes*.

a *Controversial;*

* *Study in rats*.

### Extracellular Nucleases

Apoptotic cells are rapidly cleared by tissue macrophages. This process, termed efferocytosis, plays a crucial role in the disposal of extracellular self-NAs thus limiting their pathogenic potential (149). Circulatory cfNAs are also eliminated by hepatorenal clearance mechanisms (150, 151); however, both of these regulatory processes are not sufficient and require further help from extracellular nucleases. These enzymes, comprising both DNases and RNases, play a crucial role in the regulation of the abundance, the size, and the immunostimulatory potential of endogenous cfNAs as supported by *in vivo* studies and clinical observations indicating that their deficiencies and dysregulation contribute to the development of autoimmune syndromes.

#### Extracellular DNases: Key Regulators of Cfdna-Mediated Systemic Autoimmunity

The main extracellular nucleases targeting DNA belong to the deoxyribonuclease (DNase)-1 family and include DNASE1, DNASE1like1 (DNASE1L1), and DNASE1like3 (DNASE1L3). They show a high degree of homology (~51%) and comparable structures including a DNASE domain preceded by a signal sequence that is required for their trafficking to the ER and intracellular inhibition of their DNASE activity (29). Upon trafficking to the ER the signal sequence is cleaved, allowing the secretion of fully active DNases (152, 153). Contrary to DNASE1, DNASE1L1, and DNASE1L3 have unique C-terminal domains whose function will be discussed below. In addition to their structure these extracellular DNases share common biochemical properties: they function at neutral pH, their enzymatic activity is dependent on divalent cations (Ca^2+^/Mg^2+^/Mn^2+^) and they cleave phosphodiester bonds leaving 3' hydroxy/5' phosphor (3'OH/5'-P) ends (154). Their unique functions in the regulation of endogenous cfDNA abundance and immunogenicity will be further detailed.

***DNASE1*** is expressed primarily in the kidneys, pancreas, salivary glands, stomach and the small intestine (152). It can also be detected in body fluids such as plasma and urine (155) reflecting its secreted nature. Initially DNASE1 was thought to play an important role in the degradation of DNA from nutrients in the digestive tract but its systemic distribution suggested a broader function in the regulation of extracellular cfDNA levels. DNASE1 present in the plasma is capable of digesting “naked” DNA and nucleosomal DNA in the presence of heparin and/or plasmin (152, 156, 157). Moreover, DNASE1 was shown to digest DNA originating from NETs *in vitro* as well as *in vivo* (158, 159). Its functional characterization and relevance in the regulation of extracellular cfDNA abundance and immunogenicity was assessed after the generation of *Dnase1*-deficient (KO) mice (160). These mice spontaneously developed anti-dsDNA and anti-nucleosome autoantibodies, and ultimately glomerulonephritis. With these specific pathological manifestations, *Dnase1* deficiency was suggested to cause SLE development *in vivo* (160). However, these results were obtained in mice that were on a mixed 129sv-C57/Bl6 background which are genetically predisposed to autoimmunity (161), and were not reproduced in *Dnase1* KO mice backcrossed to pure 129Sv and C57/Bl6 backgrounds (162). In addition, genetic targeting of *Dnase1* in these mice caused inactivation of the *Trap1* gene, encoding a mitochondrial chaperone, due to the location of its open reading frame on the opposite DNA strand of *Dnase1* (163). Therefore, the impact of *Dnase1* deficiency on SLE development is dependent on the genetic background and may be confounded by the unintentional *Trap1* inactivation. The levels of circulatory cfDNA and its overall size distribution was also recently shown to be similar between control and *Dnase1* KO mice (164), further supporting a redundant, rather than primary role for DNASE1 in the regulation of cfDNA abundance and immunogenicity. Interestingly, Kenny et al. have generated a new *Dnase1* KO strain on a C57/Bl6 background without affecting the expression of *Trap1*, which shows divergent results. These mice develop SLE features as manifested by elevated levels of anti-dsDNA antibodies and a mild glomerulonephritis (83). It is difficult to estimate the significance of the autoantibody titers detected in these mice in comparison to other SLE-prone mouse strains, therefore further studies are needed to clarify the role of DNASE1 in SLE and its mechanisms of action in mice. Similarly, the involvement of DNASE1 in SLE pathogenesis in humans remains unclear. Genetic studies have identified a heterozygous non-sense mutation in *DNASE1* of SLE patients (82) and single nucleotide polymorphisms (SNP) in *DNASE1* that are associated with susceptibility to SLE (165, 166). Nevertheless, follow up studies failed to shed light on mutations in *DNASE1* in numerous cohorts of SLE patients (167–171). Moreover, SLE patients were reported to exhibit a reduced circulatory DNASE1 activity (172) and such reduced activity was later associated with the development of kidney disease (158). DNASE1 activity in these studies was established by analyzing the ability of SLE patient sera to digest either naked (172) or NET-associated DNA (158), which are substrates shared with other circulatory nucleases such as DNASE1L3 (156). Finally, trials aiming to supplement SLE patients with recombinant human DNASE1 failed to show clinical benefits (173). Hence, in both humans and mice, DNASE1 doesn't seem to be a major safeguard mechanism preventing the break of tolerance to self-DNA. Rather than displaying a systemic function, numerous studies point toward a local role of DNASE1, particularly in kidneys where it may limit the pathogenic properties of immune complexes in SLE patients (174, 175).

**Figure 2:**
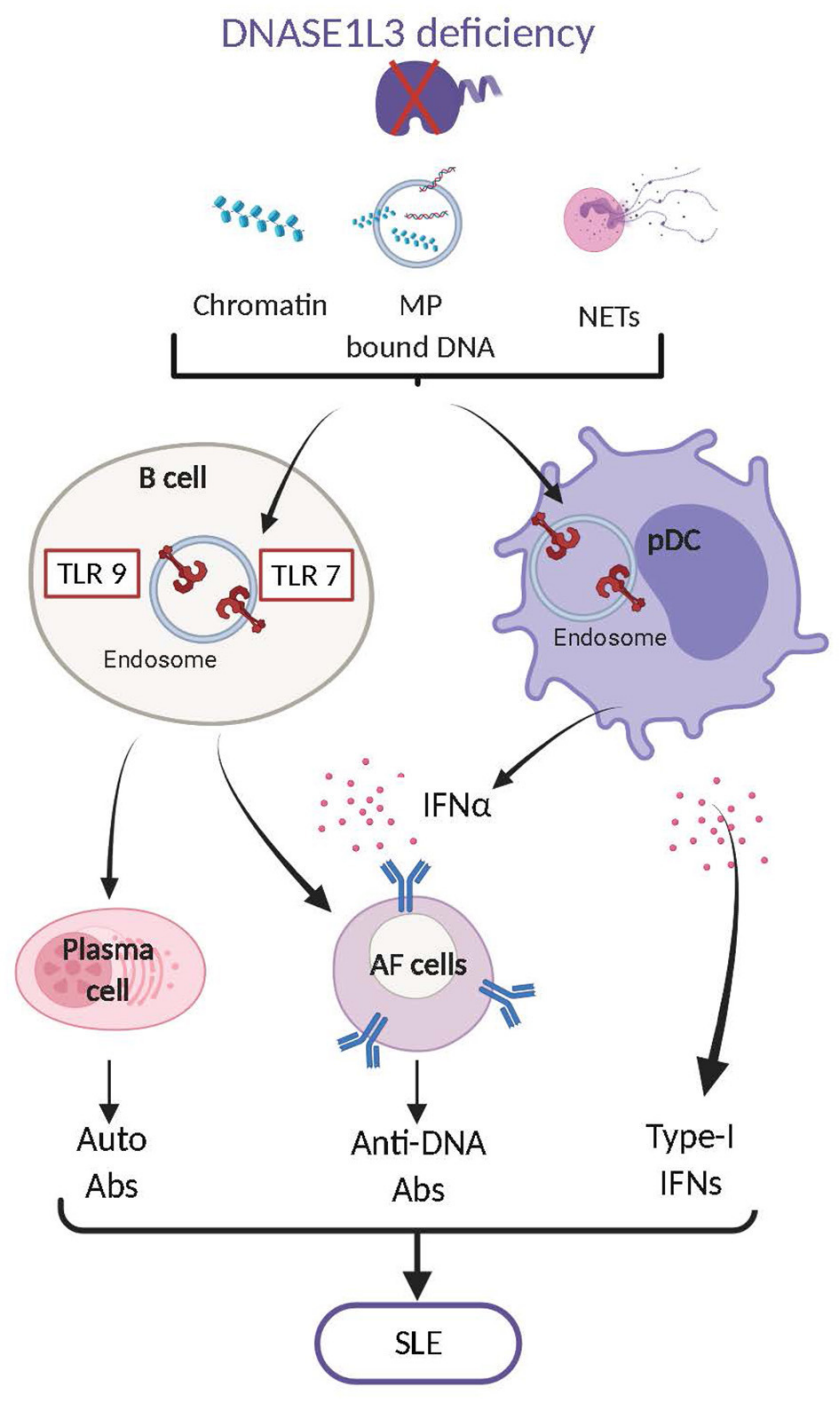
DNASE1L3 deficiency leads to the accumulation of numerous forms of DNA including chromatin, MP associated DNA and NET-associated DNA. Accumulation of such DNA contributes to the aberrant activation of TLR7,9 in B cells and plasmacytoid dendritic cells (pDCs). In B cells TLR7,9 activation leads to their differentiation into plasma cells and antibody forming cells (AFC) that produce autoreactive antibodies mostly directed against dsDNA. In pDCs TLR7,9 activation induces the production of type I interferons (IFN-I) which also play an important role in the transition of B cells into AFC. The production of anti-dsDNA antibodies and of IFN-I will ultimately cause the development of Systemic Lupus Erythematosus (SLE).

***DNASE1L3*** was initially identified in rat thymocytes (176) and later in the liver and the spleen (177, 178), pointing to a specific expression in hematopoietic cells. Further studies showed that DNASE1L3 is highly expressed in cells of myeloid origin, including DCs and macrophages (45, 179). In addition, inflammatory signals such as IL-4 were recently reported to induce DNASE1L3 expression in human myeloid cells (180). Together with DNASE1, DNASE1L3 was shown to account for most of the DNase activity measured in murine serum (156). DNASE1L3 is capable of digesting “naked” DNA and DNA in NETs, although less efficiently than DNASE1 (156, 158, 159). Besides its shared function with DNASE1, DNASE1L3 possesses a unique ability to degrade nucleosomal DNA (chromatin) without helper proteases (152, 181) and DNA encapsulated in liposomes (179), as reflected by its ability to prevent cell transfection. Given its potential to digest liposome encapsulated DNA, we investigated what may be the natural substrate of DNASE1L3 and identified DNA associated to MPs released by dying cells (45). These unique properties of DNASE1L3 rely on its C-terminal α-helical domain of 21 amino acids that is positively charged and highly hydrophobic. Deletion of DNASE1L3 C-terminus abrogates its potential to digest nucleosomal, liposome-encapsulated and MP-associated DNA without affecting its ability to degrade “naked” DNA (45, 179). The biochemical features of DNASE1L3 C-terminal domain may facilitate lipid membrane binding, penetration as well as the displacement of histones from nucleosomes. Nevertheless, the mechanisms of action of DNASE1L3 C-terminal domain are still speculative and require further investigation. Overall, DNASE1L3 regulates the abundance of numerous sources of extracellular endogenous DNA. The implications of DNASE1L3 in regulating the immunostimulatory potential of endogenous DNA came initially from human studies. Pioneering work by Al-Mayouf et al. has led to the identification of autosomal recessive homozygous null mutation in *DNASE1L3* that caused severe childhood-onset SLE (88). Additional studies followed and identified multiple families with different null mutations in *DNASE1L3* that were associated with the development of early onset SLE and SLE associated diseases (89–92). In addition, SNPs in *DNASE1L3* that cause mutations and functionally impair DNASE1L3 were reported to confer susceptibility to SLE, and related autoimmune diseases such as systemic sclerosis and rheumatoid arthritis (93–99). Altogether these results clearly indicate that DNASE1L3 regulates the potential of endogenous DNA to aberrantly activate autoimmune responses. Similar to humans, *Dnase1l3* KO mice developed anti-DNA and anti-nucleosome antibodies by 5 weeks of age, that accumulated over time and ultimately caused minor kidney pathology (45). The phenotype induced by *Dnase1l3* deficiency in mice was milder than in humans and did not result in lethality. This difference is likely due to the housing of *Dnase1l3* KO mice in specific pathogen free facilities, since their treatment with exogenous IFN-I significantly accelerated the diseases and induced lethality (45). Similar SLE features were reported in an additional strain of *Dnase1l3*-deficient mice and the autoimmune phenotype was further enhanced when associated with Fc gamma receptor IIB (*Fcgr2b*) deficiency (100). Abrogation of DNASE1L3 both in mice and humans caused the accumulation of endogenous cfDNA particularly in MPs (45). Contrary to *Dnase1 KO* mice, *Dnase1l3* KO mice (182), and *DNASE1L3* null humans (183) also display significant modifications of their circulatory cfDNA, including elevated levels of long poly-nucleosomal DNA fragments. These results clearly demonstrate an important function of DNASE1L3 in reducing the availability of immunogenic cfDNA by restricting DNA length and reducing its exposure on MPs derived from apoptotic cells. The development of SLE features in *Dnase1L3* deficient mice was STING independent but MyD88 dependent supporting the role of endolysosomal TLR in the detection of cfDNA accumulating in these mice (45). TLR7 together with TLR9 were later shown to play a crucial role in SLE development in *Dnase1l3* KO mice (101). The apparent redundancy of TLR7 and 9 in *Dnase1l3* KO may rely on the ability of TLR9 to recognize DNA and TLR7 to broadly recognize NA degradation products such as deoxyguanosines (21). Finally, TLR7/9 activation by endogenous DNA is crucial for stimulating the production of anti-DNA antibodies by B cells and induces production of IFN-I by pDCs which further “boosts” autoreactive B cell responses (101). Therefore, in both mice and humans, DNASE1L3 plays a crucial role in preventing the pathogenic activation of immune responses by endogenous DNA released by dying cells (Figure 2).

**DNASE1L1** expression is restricted to the skeletal muscle and cardiomyocytes (184, 185). DNASE1L1 contains a glycosylphosphatidylinositol (GPI) anchor located in its C-terminus that prevents its secretion (184) as reflected by its absence in body fluids. DNASE1L1 is anchored to the cell membrane with its DNase domain sticking out in the extracellular space and thus likely functions extracellularly (184). DNASE1L1 is also capable of digesting naked DNA, but its specific function remains poorly understood. Overexpression and siRNA-mediated knockdown in a human rhabdomyosarcoma cell-line demonstrated that DNASE1L1 reduced the transfection of DNA encoding a reporter protein (184). These observations suggest that DNASE1L1, similar to DNASE1L3, may degrade DNA complexed with transfection reagents, but its mechanism of action and natural substrates remain unknown. *Dnase1l1*-deficient mice displayed reduced fatigue tolerance and revealed notable evidence of damage/regeneration in muscle fibers (87), providing further evidence of its specific function in muscle tissues. Myocytes of skeletal muscle contain specialized structures, like T-tubules and caveolae, that have been proposed as entry sites for exogenous DNA and express multiple NA sensing PRR involved in the induction of muscle inflammation (186). Whether DNASE1L1 functions to protect myocytes from extracellular DNA mediated tissue inflammation requires further investigation. Supporting its role in muscle tissue, human studies have identified SNPs in *DNASE1L1* associated with the development of Pompe's disease (84), which is a metabolic disorder characterized by myopathy, respiratory weakness, physical disability and premature death. However, studies involving larger and more diverse cohorts did not validate these observations (85) and the only SNP in *DNASE1L1* that abrogates its endonuclease activity was not associated with Pompe's disease (187). Conversely, a recent analysis suggested that *DNASE1L1* SNPs may be linked to type 1 diabetes, schizophrenia and ANCA-associated vasculitis (86). Therefore, further studies of DNASE1L1 are required to understand its function and whether it also regulates the immunostimulatory potential of cfDNA.

Overall, extracellular DNases have distinct tissue distributions and functions (Table 1). While DNASE1L1 function is less understood, DNASE1 and DNASE1L3 are required in different ways for the prevention of autoimmune responses induced by cfDNA. DNASE1 deficiency does not cause severe disease nor does it alter the overall length distribution of circulating cfDNA, likely due to the presence of DNASE1L3 and primary role of this enzyme in cfDNA maintenance. It may however display tissue-specific function, i.e., in the kidney where it may alleviate the pathogenic properties of immune complexes. On the other hand, DNASE1L3 uniquely disposes of poly-nucleosomal DNA and MP-associated DNA, which otherwise contribute to SLE development both in mice and humans (Figure 2). Beyond their individual roles, DNASE1 and DNASE1L3 were recently shown to work in concert to prevent vascular occlusion induced by NETs during chronic neutrophilia and sepsis (159).

#### Extracellular RNases: Unclear Function in the Regulation of Cfrna Abundance and Immunostimulatory Potential

The potential of cfRNA to become immunostimulatory is less characterized compared to cfDNA, and so are the functions of extracellular RNases in the regulation of these processes. There are two main families of extracellular RNases, including RNASEA (also known as RNASE1) and RNASET2.

**RNASEA** is a vertebrate-specific superfamily of extracellular secreted small cationic ribonucleases expressed mostly within immune cells that share sequence similarities, a disulfide-bonded tertiary structure and the common ability to degrade ssRNA at neutral pH. The family has eight canonical members (RNASE 1-8) that have a conserved RNA-degrading catalytic domain. Additionally, there are 5 reported non-canonical members (RNASE 9-13), that are involved in male-reproductive functions but do not possess ribonuclease activity (188). Although the major function of several RNase A family members is digestion of dietary RNA, several of them have evolved to perform antibacterial, antiviral and immune modulatory functions (189). Importantly, a variety of host defense-related activities attributed to members of the RNase A family are independent of their ribonuclease function (190, 191). A role for RNASEA in the regulation of extracellular cfRNA abundance and immunostimulatory potential was reported in pathological models of hepatic and cardiac ischemia (192, 193). Supplementation of RNASEA prevented the accumulation of circulatory cfRNA released by hypoxic tissues during ischemia and its ability to induce further complications through the activation of inflammatory responses (192, 193). However, there is no clear genetic evidence yet, that RNASEA family members represent important safeguard mechanisms to avoid development of autoimmunity, likely due to their redundant activities.

**RNASET2** belongs to a family of ancient RNases whose expression and function are conserved from viruses to humans (194). In vertebrates RNASET2 is broadly expressed, digests ssRNA and functions at acidic pH (194). Although, RNASET2 localizes in lysosomes, where the acidic pH facilitates its RNA digesting activity, it can also be secreted in the extracellular milieu. RNASET2 performs a variety of functions, including modulating host immune responses and serving as extra- or intracellular cytotoxins, reviewed by Luhtala et al. (194). The latter functions of RNASET2 are either dependent or independent of its ribonuclease activity but its role in the degradation of extracellular cfRNA and regulation of RNA-mediated inflammatory responses has only been marginally explored. Loss of function mutation of RNASET2 in humans was shown to lead to the development of a neurological disease called cystic leukoencephalopathy (102). The clinical manifestations induced by *RNASET2* deficiency resulted from aberrant central nervous system inflammation, that may be induced by accumulation of either extra or intracellular immunostimulatory RNA (102). *RnaseT2* deficient rats reproduced neurological clinical features (103) providing an interesting model for the exploration of cellular and molecular mechanisms involved in this process. It would be particularly interesting to understand how the deficiency of a broadly expressed RNase preferentially affects the central nervous system.

Therefore, the function of RNases in the regulation of endogenous RNA abundance, immunostimulatory potential and their involvement in autoimmune and inflammatory disorders await further discoveries.

### Intracellular Nucleases and NA-Editing Enzymes

NAs released extracellularly can reach the intracellular space upon uptake by innate immune cells. Intracellular NAs originating from mitochondria and the nucleus can also gain access to these intracellular compartments where they can activate NA sensing PRR and an inflammatory response. Therefore, strategies to regulate the abundance of intracellular NA are crucial for avoiding harmful immune activation. Such safety measures have been selected over the course of evolution and involve intracellular nucleases and NA modifying enzymes. The control of intracellular NA levels and their immunostimulatory potential by these enzymes provides a second line of defense from NAs that escape extracellular control and the first line of defense for NAs derived from intracellular compartments. These nucleases and NA-editing enzymes can be subdivided in two main classes: those which reside in endolysosomes and control NAs internalized from the extracellular space by endocytosis and from the intracellular space by autophagy and those residing in the cytosol which control the levels of intracellular NAs. Their unique properties, functions and their involvement in the induction of tolerance to self-NA will be discussed in detail bellow and are summarized in Table 1.

#### Endolysosomal DNases Prevent Fatal Inflammatory Responses Induced by Self-DNA

**DNASE2** family is comprised of 3 members including DNASE2A, DNASE2B and Leucocyte Elastase Inhibitor (LEI) DNASEII (L-DNaseII). While DNASE2A and DNASE2B are conserved between species and share 66% homology, L-DNASEII shows only 29% homology with the two others (29). L-DNASEII is peculiar in that it is derived from post-translational modifications of LEI also called SerpinB1 (195). Due to the inability to dissociate L-DNASEII specific function from LEI, its role in the regulation of endogenous DNA is poorly characterized and thus won't be considered further in this review. On the other hand, DNASE2A and DNASE2B were extensively studied and play an important role in the control of immunogenic self-DNA. They share similar structures with a signal sequence and two phospholipase D (PLD) signature motifs in their catalytic domain. The signal sequence goes through glycosylation, which is required for their transition into active enzymes (196). The PLD motifs form a single active site containing histidines which are essential for their catalytic function (197, 198). Furthermore, both DNASE2A and 2B harbor conserved mannose phosphorylation motifs involved in their transport into endolysosomes (199). They cleave dsDNA into short oligonucleotides bearing 3'-P rather than 3'-OH ends. The activity of these enzymes is independent of divalent cations and is optimal at acidic pH, found in endolysosomal compartments (195). Although they share many similarities, DNASE2A and DANSE2B have different tissue expression profiles and their dysregulation or deficiency induces different consequences *in vivo*.

**Figure 3:**
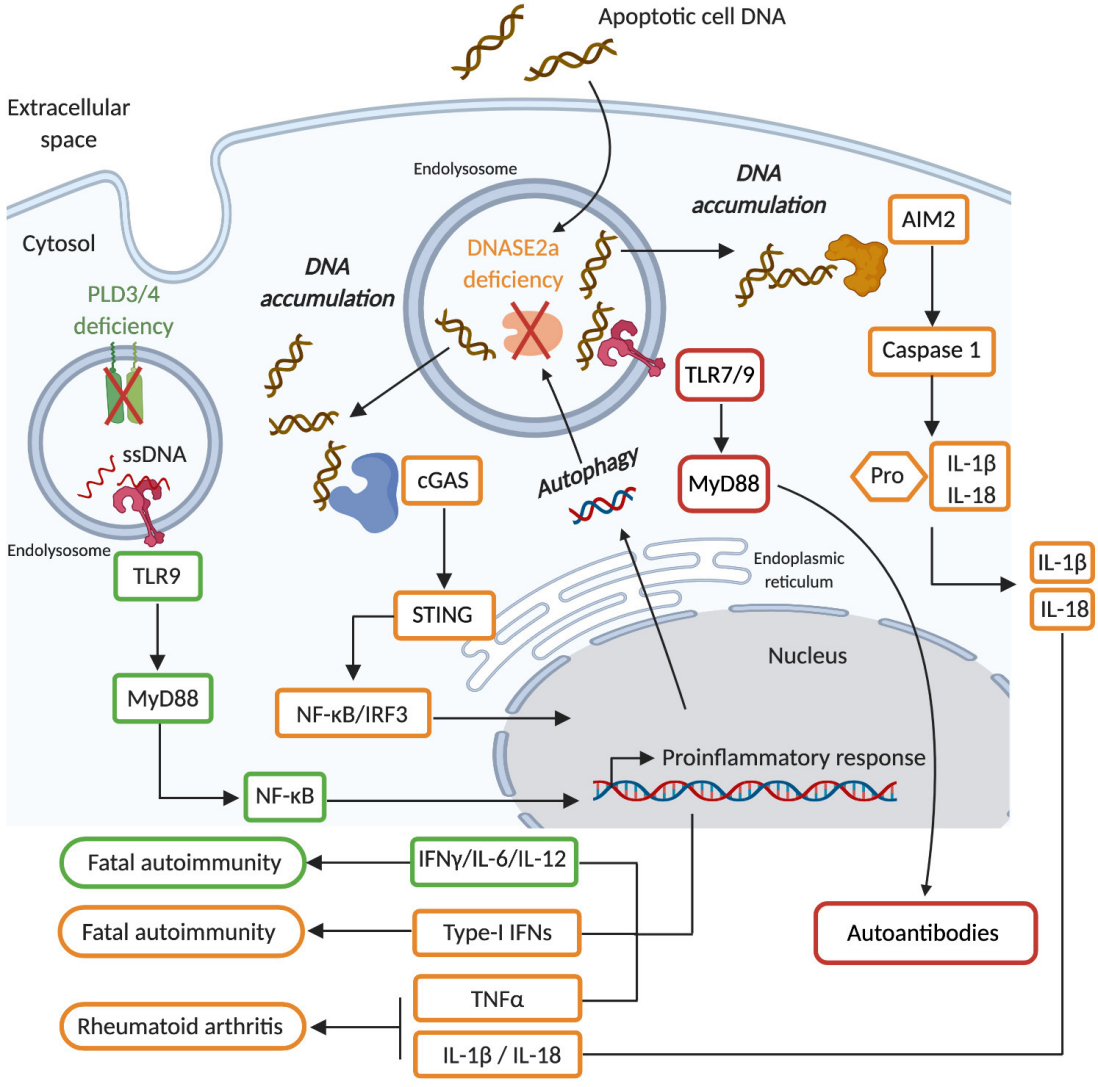
DNASE2A deficiency causes an accumulation of phagocytosed dsDNA as well as nuclear dsDNA transported by autophagy into endolysosomal compartments. Such dsDNA can gain access to the cytosol and activate the cGAS/STING pathway (orange) that leads to the production of type I interferons (IFN-I), which ultimately causes the development of fatal autoimmunity. In the absence of IFN-I signaling DNASE2A deficiency will induce the development of rheumatoid arthritis which is mediated by TNFα, whose production is triggered by the cGAS/STING pathway, but also by IL-1β and IL-18 which are inflammatory cytokines produced upon the activation of the AIM2 inflammasome by cytosolic self-DNA. Finally, DNASE2A deficiency also induces aberrant activation of endolysosomal TLR (red) which contribute via the activation of the MyD88 to the production of autoantibodies. PLD3/4 are novel endolysosomal nucleases (green) involved in the degradation of ssDNA. Their deficiency induces an accumulation of ssDNA in endolysosomes that ultimately activates TLR9-MyD88-NFκB mediated inflammatory cytokine (IL-6 and IL-12) production, causing fatal autoimmunity.

*DNASE2A* is highly expressed in macrophages that are present in most tissues. Initial observations that inhibition of macrophage endolysosomal acidification blocked the fragmentation of engulfed DNA from apoptotic cells, suggested that DNASE2A plays an important role in the disposal of DNA from apoptotic cells (200, 201). This function was confirmed in *Dnase2a* KO mice in which DNA-containing bodies (DCB) originating from apoptotic cells accumulated in multiple organs early in development (107). The highest number of DCB was present in the liver and attributed to the impaired ability of fetal liver macrophages to dispose of extruded nuclei from erythrocytes (107, 108). Consequently, *Dnase2a* KO mice exhibited severe anemia and inflammation caused by the improper clearance erythrocytes' nuclei, that ultimately induced lethality at an early stage of mouse development (E17.5) (108, 202). In addition, thymic development of T cells was severely impaired in *Dnase2a* deficient mice due to poor elimination of apoptotic thymocytes and subsequent inflammation (203). To avoid embryonic lethality and study *Dnase2a* function in adult mice, BM chimeras were established and *Dnase2a*-floxed animals were generated. Mice reconstituted with *Dnase2a* deficient BM cells developed severe chronic polyarthritis, indicating that specific deletion of *Dnase2a* in hematopoietic cells regulates the ability of endogenous DNA to cause arthritis (109). Similar results were obtained in *Dnase2a*-floxed animals crossed with the *Mx1*-cre strain, in which *Dnase2a* was abrogated in all IFN-I responsive cells following poly(I:C) treatment (110). Interestingly, the conditional deletion of *Dnase2a* caused an accumulation of DNA in multiple cell lineages beyond macrophages including T cells, B cells and fibroblasts, suggesting a broader function of DNASE2A (204, 205). In this context DNASE2A was proposed to function in a cell-autonomous manner and to regulate DNA originating from the nucleus and accumulating in autophagosomes (204, 205). Embryonic lethality induced by *Dnase2a* deficiency was attributed to an aberrant production of IFN-I, since *Dnase2a-Ifnar1* double-KO mice were healthy for at least 8 weeks post birth (206). The sensing of DNA causing this IFN-I production in *Dnase2a* deficient mice was shown to be independent of endosomal TLRs (207) but fully dependent on the cGAS-STING pathway (112, 113). These results suggested that DNA from engulfed apoptotic cells and/or that accumulates in a cell autonomous manner, can exit from endolysosomal compartments into the cytosol in the absence of DNASE2A to stimulate cGAS-STING-mediated IFN-I production. Interestingly, while *Dnase2a-Tmem173* (gene encoding STING) double KO mice were healthy (112, 113) *Dnase2a-Ifnar1* double-KO mice develop rheumatoid arthritis that was driven by TNFα (Tumor Necrosis Factor-α) (109, 110). Therefore, aberrant activation of the cGAS-STING pathway in the absence of DNASE2A also contributed to polyarthritis development by inducing the production of TNFα. Furthermore, *Aim2* deletion added to *Dnase2a-Ifnar1* double-deficiency ameliorated the polyarthritis phenotype in these mice, indicating AIM2 inflammasome activation in the absence of DNASE2A contributes as well to this disease (114, 115). *Dnase2a-Ifnar1* double-KO mice develop elevated levels of autoreactive antibodies directed against nuclear material as they age (114). Surprisingly, this production of anti-nuclear antibodies was independent of STING and AIM2 but fully dependent on endolysosomal TLRs (114). Thus, DNA that accumulates in *Dnase2a* deficient mice activates most of the DNA sensing pathways that then differentially contribute to pathological features. These observation in mice were corroborated in human studies by the identification of individuals with null mutations in *DNASE2A* that show severe non-regenerative anemia and deforming arthropathy (104). *DNASE2A* deficiency was accompanied by an up-regulation of interferon-stimulated genes (ISGs) and elevated TNFα levels, suggesting similar pathogenic pathways at play in these patients as in *Dnase2a* KO mice (104). In addition, SNPs in *DNASE2A* were associated with rheumatoid arthritis (105, 110) and revealed weak association with the risk of renal pathology in SLE patients (106). Therefore, in both mice and humans, DNASE2A is critical for eliminating self-DNA and limiting its capacity to induce harmful inflammatory and autoimmune responses (Figure 3).

*DNASE2B* expression is restricted to lens cells where it plays a key role in degrading fiber cell nuclei to regulate their differentiation (116). Accordingly, *Dnase2b* KO mice present undifferentiated fiber cells containing condensed undigested DNA leading to the development of cataracts (116). The expression of *Dnase2b* is regulated by lens-specific heat shock transcription factor 4 (HSF4) (208, 209), the deficiency of which in mice caused cataracts and SNPs in humans were linked to cataractogenesis (210–212). Unlike DNASE2A, DNASE2B is relocated from the endolysosomal compartment to the nucleus of lens cells where it degrades nuclear DNA (213). DNASE2B transport to the nucleus is mediated by cyclin-dependent kinase 1 (CDK1) inhibitor p27^kip1^ whose specific deletion in the lens delays the de-nucleation of lens fiber cells (214). Despite DNA accumulation induced by DNASE2B deficiency, no inflammation was observed in the lens or other tissues of these animals (116). This is likely due to the specific expression of DNASE2B in the eye which is an immune-privileged site that expresses low levels of proteins involved in NA sensing pathways. Thus, DNASE2B displays a cell-autonomous function to allow differentiation of functional lens fiber cells by degrading their nuclear material (209).

**Phospholipase D (PLD)** are a family of enzymes that are broadly expressed and whose function is conserved from bacteria to mammals. They comprise 4 members (PLD1-4). PLD1 and PLD2 catalyze phosphatidylcholine into choline and phosphatidic acid. In contrast, PLD3 and PLD4 are non-classical PLDs that lack phospholipase D activity (215, 216), but contain an N-terminal transmembrane domain allowing their localization in endolysosomes (217, 218). While rare coding variants of *PLD3* were associated with Alzheimer's disease (219, 220) and SNPs in *PLD4* conferred susceptibility to rheumatoid arthritis and systemic sclerosis (117–119), their function remained largely unknown until recently. Gavin et al. have shown that PLD3 and PLD4 are 5' exonucleases that degrade ssDNA at both neutral and acidic pH, which is compatible with their endolysosomal localization (120). Hence *Pld4* KO mice showed signs of chronic inflammation including splenomegaly and aberrant innate immune cell activation which were dependent on IFNγ and TLR9 but independent of T and B cells (120). Conditional deletion of *Pld4* in DCs but not in macrophages showed a similar phenotype, indicating that TLR9 activation by endogenous ssDNA in DCs is mostly responsible for the inflammation induced by *Pld4*-deficiency (120). On the other hand, *Pld3* KO mice did not show inflammatory manifestations of *Pld4* KO mice, but macrophages from *Pld3* KO mice produced elevated levels of proinflammatory cytokines in response to TLR9 stimulation (120). DCs express *Pld4* and macrophages express *Pld3* and they exhibit functional redundancy since *Pld3-Pld4* double deficient animals did not survive longer than 21 days due to severe liver inflammation (120). Thus, PLD3 and PLD4 are novel nucleases working together in the endolysosomes of innate immune cells to prevent endogenous ssDNA sensing by TLR9 and thus the development of inflammatory syndromes (Figure 3), but further investigation is needed to address the relevance of PLD3/4 in humans and how they may contribute to specific autoimmune disorders (117–119).

**DNASE1L2** belongs to the DNASE1 family and shares similar structure and properties with other family members described above, except that DNASE1L2 functions optimally at acidic pH (154). The fact that DNASE1L2 degrades its substrates at acidic pH, suggests an endolysosomal and/or autophagosomal localization of this enzyme. DNASE1L2 is specifically expressed in keratinocytes and participates in their differentiation into corneocytes by degrading their nuclei (221). *Dnase1l2* deficient mice, showed a retention of nuclear DNA (parakeratosis) in the hair, nails and other epithelial tissues but did not show any skin defect (122) suggesting that DNASE1L2 function in skin keratinocytes may be compensated. It was indeed the case, and DANSE1L2 was reported to work together with DNASE2A in the disposal of nuclear DNA from skin keratinocytes (222) and with the exonuclease TREX2 in the degradation of DNA from lingual keratinocytes (223). Nevertheless, *Dnase1l2-Dnase2a* and *Dnase1l2-Trex2* keratinocyte specific double deficient animals generated in these studies did not develop signs of epithelial tissue inflammation despite the accumulation of DNA (223). The inability of DNA to aberrantly activate inflammatory responses in this instance is likely due to the low expression of NA sensors in keratinocytes (223) as well as the formation of a cornified envelope that physically prevents DNA from reaching epidermal and dermal compartments rich in innate immune cells. Observations in humans however show that SNPs in *DNASE1L2* are associated with psoriasis (121) and that *DNASE1L2* expression is reduced in the inflamed psoriatic skin (221). These observations suggest that DNASE1L2 in humans may somehow regulate skin inflammation but requires further investigation to understand its mechanism of action.

It appears that endolysosomal DNases expressed by innate immune cells are of paramount importance to prevent the development of severe inflammatory disorders induced by the accumulation of intracellular self-DNA (Table 1). Particularly DNASE2A disposes of self-dsDNA and prevents its ability to activate most NA sensing pathways, while PLD3/4 degrade self-ssDNA capable of aberrantly activating TLR9 (Figure 3). Conversely, DNASE2B and DNASE1L2 are tissue specific nucleases involved in the degradation of nuclear DNA of lens cells and keratinocytes, respectively. Their primary function is thus to contribute to tissues development, while their role in the regulation of immunostimulatory DNA seems secondary or even non-existent. Finally, endolysosomal RNases are poorly characterized and will not be discussed in greater detail here. RNASET2, which was previously discussed, can be found in endolysosomes (194), but its function in the regulation of RNA immunostimulatory potential remains poorly characterized.

#### Cytosolic Nucleases and NA-Editing Enzymes Prevent IFN-I-Mediated Syndromes (Interferonopathies)

**Figure 4:**
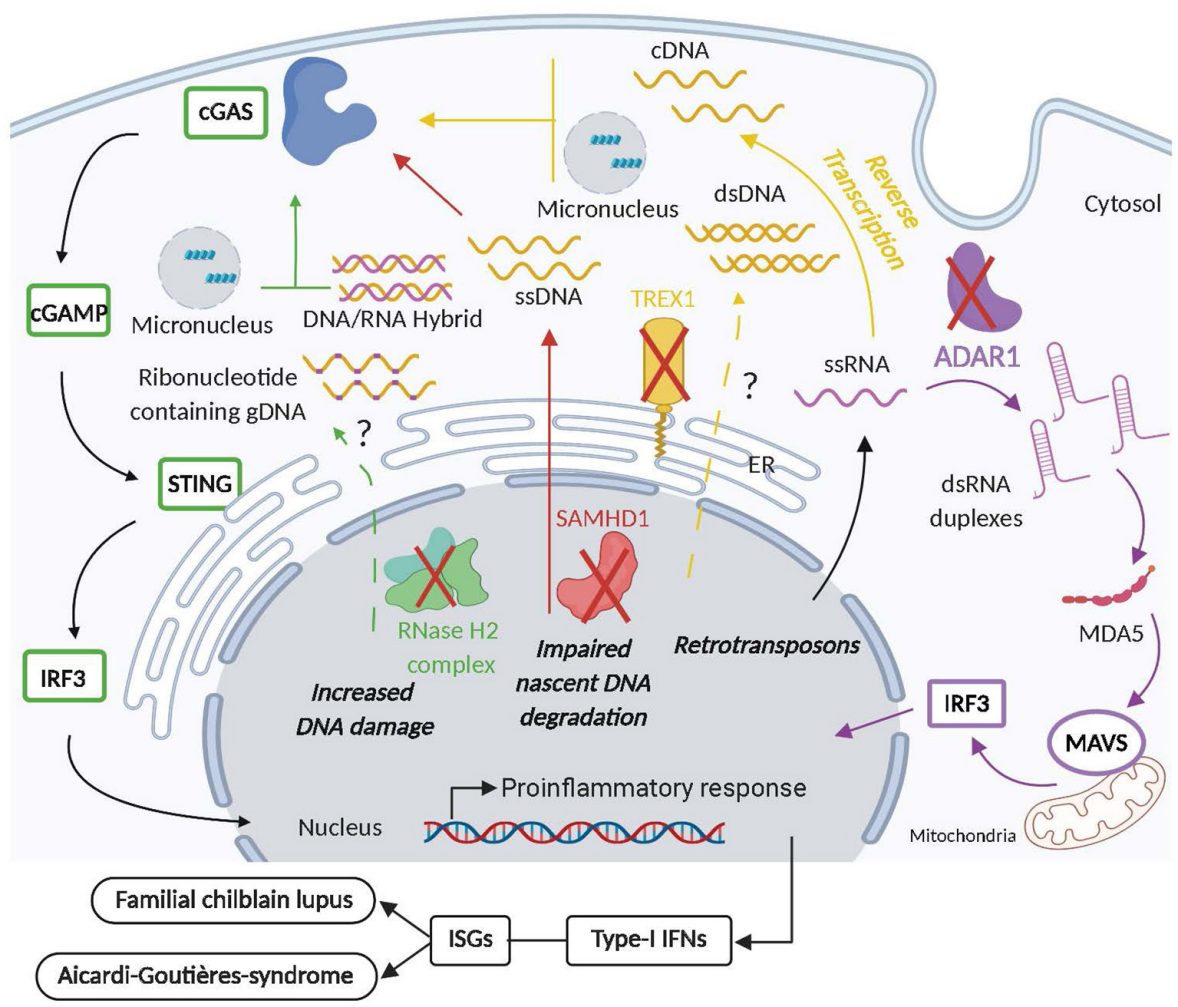
Intracellular nuclease and NA-editing enzyme deficiency aberrantly activates inflammatory and autoimmune responses. Mutations in TREX1 (yellow) that affect its exonuclease activity or cause its deficiency, lead to the cytosolic accumulation of ssDNA originating from the reverse transcription of endogenous LTR-retrotransposons that activate the cGAS/STING pathway causing the secretion of pathogenic type-I interferons (IFN-I) and the development of familial chilblain lupus (FLC) and Aicardi Goutières Syndrome (AGS). Furthermore, dsDNA and micronuclei may be detected in the cytosol of individuals with dysfunctional TREX1 and contribute the aberrant activation of the cGAS/STING pathway as well. Mutation in SAMHD1 (red) causes the release of ssDNA from stalled replication forks that stimulates IFN-I production upon the activation of the cGAS/STING pathway that may ultimately contribute to AGS and FCL. AGS is also associated with mutations in RNase H2 complex (green) that reduces ribonucleotide excision repair (RER) and thus increases DNA damage and possibly the release of DNA:RNA hybrids, micronuclei and ribonucleotide containing gDNA into the cytosol that may activate cGAS/STING-mediated production of deleterious IFN-I. Finally, dysfunction in ADAR1 (purple) leads to the accumulation of dsRNA duplexes originating from endogenous retrotransposon RNA in the cytosol which upon activation of the MDA5 pathway causes the secretion of pathogenic IFN-I responsible for AGS development.

**TREX1** (Three Prime Repair Exonuclease 1), also called DNASE3, is a broadly expressed 3' → 5' exonuclease that is associated with the ER at steady state, and re-localizes into the nucleus after DNA-damage (224, 225). TREX1 contains an N-terminal domain with an exonuclease activity and a C-terminal transmembrane domain allowing its attachment to the ER and regulating its overall activity (226–228). Its exonuclease domain is key for DNA digestion and it has been shown to dispose of ssDNA and dsDNA (229, 230). Initially TREX1 was described to play a role in DNA repair upon its translocation to the nucleus (132, 226), but observations that *Trex1* deficient mouse embryonic fibroblasts (MEFs) accumulated ssDNA in the ER following induction of DNA damage showed that TREX1 is essential for ssDNA degradation (224, 225). TREX1 was also recently ascribed novel functions independent of DNA degradation that mostly rely on its C-terminal domain. Indeed, TREX1 was reported to control the biogenesis of endolysosomal compartments (131) and, through an interaction with the ER resident oligosaccharyltransferase (OST), to regulate the production immunogenic free glycans (129). The generation of *Trex1* deficient mice demonstrated that its main function is to prevent the development of aberrant inflammatory immune responses. *Trex1* KO mice did not show excessive DNA damage but displayed severe inflammatory myocarditis, systemic inflammation and elevated titers of autoantibodies, causing their premature death (81, 130). *TREX1* loss of function mutations in humans were also associated with the development of Aicardi-Goutières-syndrome (AGS) (123). AGS is an inflammatory syndrome characterized by an encephalitis accompanied by lymphocyte infiltration in the central nervous system and an IFN-I signature (124). Furthermore, different mutations affecting *TREX1* in humans were also shown to be associated with SLE pathogenesis (125, 126) and to cause monogenic familial chilblain lupus (FCL), an inflammatory pathology manifested by ulcerating lesions of the skin (124, 127). Some AGS patients also show characteristic skin lesions similar to those of patients with FLC. Most of the mutations associated with AGS, SLE, and FCL are localized in the N-terminal exonuclease domain of TREX1 (231), suggesting an important function of TREX1 in the disposal of potentially immunostimulatory endogenous DNA. The phenotypes induced by the loss of TREX1 are different between mice and humans in that mice do not show neurological manifestations (80). Nevertheless, *Trex1* KO mice provided an excellent tool to further characterize the mechanisms through which TREX1 may control aberrant immune activation. Fatal autoimmunity in *Trex1* KO was rescued by the deletion of the cGAS-STING pathway (80, 81, 113, 132, 232), indicating that absence of TREX1 results in aberrant activation of DNA sensing pathways. ssDNA that is originating from the reverse transcription of endogenous retroelements was proposed the be the main source of endogenous DNA that accumulates upon *Trex1* deficiency and activates the cGAS-STING pathway (81). In agreement with these observations, fibroblasts from AGS patients with *TREX1* mutations showed elevated cytosolic DNA levels originating from endogenous retroelements (233). However, inhibition of reverse transcriptase in *Trex1* KO mice did not protect them from disease (234), suggesting that additional sources of endogenous DNA may contribute to the pathology observed in these mice. These additional sources of endogenous DNA may include dsDNA (235, 236) and micronuclei (237, 238) that were recently reported as TREX1 substrates. Interestingly, *Trex1*-deficient mice showed elevated levels of free glycan which is reminiscent of the ability of TREX1 C-terminal domain do regulate OST activity. Inhibition of OST ameliorated inflammatory manifestations and prolonged the survival of *Trex1* KO mice, indicating that free glycan contributes to aberrant immune activation in these mice (129). Given that free glycans act independently of the cGAS-STING pathway (129), it is difficult to reconcile these results with the observation that cGAS-STING deletion fully protects *Trex1* KO mice from disease (80). Furthermore, *TREX1* C-terminal domain mutations were also identified in humans but were not associated with AGS nor with FCL, but rather with retinal vasculopathy with cerebral leukodystrophy (RVCL) (128). Hence, how TREX1 mediated regulation of free glycans contributes to pathologies associated with TREX1 deficiency requires further investigation. At the cellular level, *Trex1* deficiency in non-hematopoietic cells was proposed to initiate inflammatory pathogenesis as *Trex1* KO mice reconstituted with WT BM cells developed inflammatory disease (80). *Trex1* KO host cells were shown to produce high levels of IFN-I that signals through IFNAR on hematopoietic cells and ultimately causes the disease (80). Nevertheless, *Trex1* KO BM cells were also shown to induce an inflammatory disease when transferred to WT recipients (239, 240), and conditional ablation of *Trex1* in DCs (CD11c-cre) and hematopoietic cells (Cx3CR1-Cre and Tie2-Cre) caused premature death (240). Therefore, both hematopoietic and non-hematopoietic cells are important sources of pathogenic IFN-I induced by *Trex1* deficiency. Finally, T cells and B cells were shown to contribute to the inflammatory pathogenesis mediated by *Trex-1* deficiency, since their individual deletion prolonged survival in *Trex1* KO mice and their combined deficiency fully rescued these mice from mortality (80). Thus, TREX1 is an essential enzyme preventing cytosolic DNA accumulation and its ability to activate cGAS-STING-mediated IFN-I production that ultimately causes fatal inflammatory syndromes (Figure 4). TREX1 deficiency differentially affects humans and mice, likely due to species-specific expression profiles of TREX1 and the fact that TREX1 is globally abrogated in mice while human mutations may only lead to partial dysfunction of the enzyme. Accordingly, when the D18N mutation found in FCL patients was knocked into (KI) mice, it induced a milder disease than*Trex1* deficiency with multiple similarities to human FCL patients including systemic inflammation, production of autoantibodies and kidney disease (235). Interestingly, the FCL-like pathogenesis in *TREX1* D18N KI mice was due to failed degradation of dsDNA (235), rather than of ssDNA as described in *Trex1* KO mice (81). Hence, investigating human TREX1 mutations in murine models *in vivo*, may be of particular relevance to better understand its pathogenic functions.

**RNases and NA editing enzymes**, within the nucleus and the cytosol are essential for the limiting the immunostimulatory potential of several endogenous NAs, and thus protect the host from the development AGS and IFN-I mediated inflammatory pathologies, as summarized in Table 1.

*The RNase H2 complex* belongs to the ribonuclease H family of ubiquitously expressed enzymes that cleave the RNA in RNA-DNA hybrids that form during replication and repair in a non-sequence-specific manner, or cleave the phosphodiester bond 5' of a single ribonucleotide embedded within a DNA duplex. The RNase H2 complex is comprised of three proteins: the catalytic subunit RNASEH2A, and subunits RNASEH2B and RNASEH2C, which do not harbor catalytic activity but are necessary for the overall function of RNase H2 complex, including its translocation from the cytosol to the nucleus (241, 242). Biallelic loss-of-function mutations in any of the three RNase H2 subunits cause AGS (133). RNase H2 enzyme complex initiates the process of ribonucleotide excision repair (RER) by removing ribonucleotides from gDNA which have been incorrectly incorporated by replicative polymerases, and hence is an integral part of the genome surveillance machinery (243, 244). This function is essential in higher eukaryotes, as loss of RNase H2-mediated RER renders gDNA susceptible to DNA strand breaks. Indeed, RNase H2 deficient mice are embryonic lethal due to a p53-dependent DNA damage response and cell cycle arrest. Not surprisingly, biallelic null mutations of RNase H2 in humans have not yet been reported. Heterozygous mutations in RNase H2 complex were shown to be associated with SLE (134) and hypomorphic *RNASEH2A/B/C* mutations were reported to cause AGS (132), both of which are enough to enhance levels of embedded ribonucleotides in gDNA (242). The ensuing sub-lethal DNA damage induces a chronic DNA damage response characterized by a heightened IFN-I response to UV light-induced thymidine-dimers (134). Using KI mouse models of human *RNASEH2A* and *2B* missense mutants detected in AGS patients, the induction of IFN-I and ISGs was found to be dependent on reduced RER and increased stimulation of the cytosolic cGAS-STING DNA sensing pathway (135, 136). Although RNase H2 mutant mice do not recapitulate the pathological features of human type-I interferonopathies, they provide compelling evidence that in the absence of a functional RNase H2 complex, endogenous NAs accumulate in the cells and are improperly sensed as non-self, leading to induction of IFN-I-mediated immune responses (Figure 4). RNase H2 dysfunction causes DNA damage due to failure to remove embedded ribonucleotides from gDNA (135, 243, 245), with micronuclei formation as one of the consequences (72). Micronuclear DNA (72, 246) as well as cytosolic RNA-DNA hybrids (247) have been shown to activate cGAS, however, the exact chemical nature of NAs stimulating the cGAS-STING pathway *in vivo* in absence of functional RNase H2 complex needs further investigation.

*SAMHD1* [Sterile alpha motif (SAM) domain and Histidine-aspartate (HD) domain-containing protein 1] is a deoxynucleotide triphosphohydrolase that is mostly expressed within immune cells. It prevents viral infections in macrophages and DCs by hydrolyzing the intracellular pool of deoxynucleotide triphosphates (dNTPs) into 2' deoxynucleoside and inorganic phosphates, thereby blocking reverse transcription of the viral genome (248). Moreover, it is upregulated through IFN-I in a MyD88-independent manner (137). Therefore, it can be surmised that dysfunction of SAMHD1 would lead to an increase in intracellular dNTP pools, thereby promoting viral replication. Interestingly, apart from *TREX1* and RNase H2, a SNP array genome-wide scan of several AGS patients and families revealed homozygous mutations in the *SAMHD1* gene, identifying a monogenic cause of AGS (137). In addition to typical AGS, dominant inheritance of a heterozygous mutation in SAMHD1 causes FCL (138). Indeed, it was subsequently shown that increased dNTP concentrations due to SAMHD1 deficiency cause genome instability, constitutive DNA-damage signaling, cellular senescence and upregulation of ISGs (249). Similarly, in mice, the absence of *Samhd1* triggers spontaneous IFN-I production and upregulation of ISGs in various cells types, however, pathological AGS-like symptoms or any inflammatory features are absent (139, 140). These studies establish SAMHD1 as a negative regulator of IFN-I signaling across species, but IFN-I production induced by *Samhd1* deficiency in mice is not sufficient to induce the development of inflammatory disorders. Although the mechanism(s) of SAMHD1 are still under investigation, it has been postulated that the excessive availability of dNTPs in the absence of SAMHD1 allows for the generation of aberrant immunostimulatory DNA intermediates which potentially trigger IFN-I response through DNA sensors (250). Additionally, recent studies have uncovered a novel function of SAMHD1 as a DNA-ds break repair enzyme working in a complex with C-terminal binding protein 1-interacting protein (CtIP) and the exonuclease meiotic recombination 11 (MRE11) (251). Mutations in SAMHD1 outside of its dNTPase activity site were found to prevent degradation of nascent DNA at stalled replication forks. The ssDNA which accumulated in the cytosol due to this defect caused aberrant activation of IFN-I signaling via the cGAS-STING pathway (252) (Figure 4).

*ADAR* (adenosine deaminases that act on RNA) are a family of broadly expressed post-transcriptional RNA editing enzymes catalyzing adenosine (A) deamination to create inosine (I) in highly structured dsRNA in a non-sequence specific manner. Most polymerases recognize inosine as a guanosine, thus by changing the primary sequence information in an RNA, ADARs generate new protein isoforms. In addition, because inosine base-pairs with cytidine, ADARs can change the structure of an RNA by changing an AU base-pair to an IU mismatch (253). In addition to its role in generating functional protein diversity, ADAR is a crucial negative regulator of IFN responses. ADAR1 deletion in mice causes embryonic lethality at E11.5–E12.5, accompanied by liver pathology, global upregulation of type I and II IFN–inducible transcripts and rapid apoptosis of hematopoietic cells (142). Not surprisingly, *ADAR1* null humans are unknown, whereas mutations in *ADAR1* cause AGS, with typical IFN-I signature (141). The conclusive evidence affirming the direct negative regulatory role of ADAR1 in the IFN-I pathway came from mouse studies, wherein conditional ADAR1 deficiency in hematopoietic cells caused a global overexpression of ISGs (141). Furthermore, the death of ADAR1 RNA editing-deficient mice (*Adar1*E861A) at embryonic day 13.5, was rescued by concurrent deletion of the cytosolic sensor of dsRNA, MDA5 (143). Similarly, ablation of MAVS, the downstream adaptor of MDA5 and RIG-I, rescued ADAR1-null mice to birth, overall demonstrating a suppressive function of ADAR1 in the RLR pathway (144). It is known that about half of the human and mouse genome is composed of non-coding retroelements such as SINEs and Alu-repeats, which typically form dsRNA duplexes. Importantly, retroelements are known substrates for extensive A-to-I RNA editing (254, 255). Indeed, genome-wide analysis of the *in vivo* substrates of ADAR1 identified clustered hyper-editing of long dsRNA stem loops within 3′ untranslated regions of endogenous transcripts, while in the absence of ADAR1 editing, long dsRNA stem loops formed that activated MDA5 (143). Overall, it is speculated that in the absence of ADAR1, unedited dsRNA transcripts originating from endogenous retroelements accumulate and activate MDA5 to induce IFN-I signaling (Figure 4). Thus, the primary physiological function of ADAR1 is to edit endogenous dsRNA to prevent sensing of this substrate as non-self by MDA5 and subsequent IFN-I response.

*RNA exosome* is a multimeric protein complex that is found in all cells and plays an essential role in the degradation of endogenous RNA. Superkiller viralicidic activity 2-like (SKIV2L) helicase which is part of the RNA exosome was recently proposed to play an important role in preventing the ability of endogenous RNA to activate IFN-I responses. shRNA-mediated knock down of *Skiv2l* in BM-derived macrophages increased RLR mediated IFN-I stimulation. Reduction of *Skiv2l* expression also induced IFN-I production in macrophages once the unfolded protein response was stimulated in a MAVS dependent manner. Furthermore, individuals with hypomorphic *SKIV2L* variants showed an elevated IFN-I signature (146). Therefore, RNA exosome through SKIV2L may negatively control endogenous RNAs to prevent activation of RLR mediated IFN-I response and thus the development of interferonopathies. Nevertheless, further investigation is needed to identify endogenous RNAs regulated by SKIV2L and its relevance in the control of the immunostimulatory potential of endogenous RNA *in vivo*, since *SKIV2L* hypomorphic mutations in humans are associated with tricohepatoenteric syndrome a rare congenital bowel disorder (145) and not severe autoimmunity.

Overall cytosolic TREX1, RNase H2 and NA-editing enzymes regulate the potential of endogenous DNA and RNA to causes inflammatory and autoimmune disorders (Table 1, Figure 4). Their dysfunction in mice and humans induces severe interferonopathies caused by an aberrant stimulation of intracellular NA sensors by endogenous NAs.

#### New Players in the Regulation of Mitochondrial Nucleic Acids

**Endonuclease G** belongs to the family of DNA/RNA-non-specific nucleases that are located in the mitochondrial intermembrane space (256, 257). MEFs deficient for endonuclease G show increased levels of ROS and elevated levels of mtDNA in the cytosol, indicating that this enzyme may regulate mtDNA abundance. In addition, mtDNA that accumulates in endonuclease G deficient MEFs was shown to induce the expression of multiple ISGs in a cGAS-STING dependent manner. mtDNA in the absence of endonuclease G accumulates into the intermembrane space of mitochondria and ultimately reaches the cytosol through voltage-dependent anion channel (VDAC) (148). SLE patients were shown to accumulate mtDNA in their circulation mostly originating from NETs (51, 61) and such accumulation of mtDNA was associated with reduced endonuclease G activity (148). Blockade of mtDNA transport to the cytoplasm using VDAC inhibitors reduced the amount of circulatory mtDNA and ameliorated lupus-like symptoms in SLE prone mice (148). Therefore, endonuclease G is an important regulator of mtDNA abundance and prevents its potential to active IFN-I production.

##### SUV3 and PNPase: Novel Regulators of mtdsRNA

Cellular RNA degradation is known to be mediated by protein complexes in specific subcellular granules such as processing bodies (P-bodies), found in the cytoplasm of eukaryotes (258). Moreover, it was shown that human mtRNA degradation is mediated by the mitochondrial RNA degradosome comprising an RNA-helicase (hSuV3) and a polynucleotide phosphorylase (PNPase), that occur in distinct foci within the mitochondria. Indeed, silencing of PNPase and/or hSuv3 caused accumulation of undegraded mtRNA decay intermediates (259, 260). More recently, the mtRNA degradosome machinery was characterized *in vivo* and the clinical and physiological relevance of mtdsRNA degradation was presented (79). A hepatocyte-specific PNPase1 deficiency caused modest increases in IFN-β and ISGs. Accordingly, patients carrying hypomorphic mutations in *PNPT1*, which encodes PNPase, displayed mtdsRNA accumulation coupled with upregulation of ISGs and other markers of immune activation, underscoring the importance of preventing cytosolic sensing of mtdsRNA for which the MDA5-MAVS pathway of cytosolic RNA sensing was required but TLR3 signaling was dispensable (79).

## Role of Nucleases in Promoting Innate Immune Responses and NA Sensing

Nucleases not only prevent accumulation of immunogenic ligands but also promote their generation. *Dnase2a* deficiency causes pathogenic activation of the cytosolic cGAS-STING pathway and autoinflammation, whereas it prevents processing of CpGA-DNA and consequently abrogates TLR9 activation in DCs. Accordingly, CpG-DNA that was pre-processed by DNASE2A was able to stimulate inflammatory cytokine production in *Dnase2a* deficient DCs (261). In addition, *Dnase2a* deficient B cells were also unresponsive to TLR9 activation by DNA complexed immunoglobulins (111). Thus, DNASE2A seems to positively regulate endosomal DNA sensing and negatively regulate cytoplasmic DNA-sensing which may be dependent on the size of DNA fragments generated by DNASE2A-dependent processing. Similarly, in addition to their role in restricting self-RNA/DNA sensing, as discussed above, intracellular RNases are also involved in generating immunogenic RNA ligands necessary for responses against pathogenic RNA. Recently, two endolysosomal enzymes RNASE2 and RNASET2 were shown to cooperatively process RNA to release uridine from RNA ligands which promote stimulation of TLR8 and provide protection against pathogenic microorganisms (262, 263). Interestingly, in addition to processing NA in order to create better ligands for PRR, nucleases may play broader regulatory functions. Particularly DNASE1L3 was reported to regulate the activation of inflammasomes. Indeed, DNASE1L3 inhibition *in vitro*, significantly altered NLRP3 and NLRC4 mediated IL-1β production upon activation (264). However, the importance of this regulatory loop *in vivo* awaits further investigation.

## Role of Nucleases in Cancer

It is becoming evident that the activation of anti-tumor immune responses is intimately connected with the activation of NA sensing pathways (265). Ablation of cGAS and STING prevented the activation of spontaneous anti-tumor immune responses (266), and suppressed the immunogenic potential of cytotoxic treatments such as radiotherapy (267) and chemotherapy (268). Similarly, TLR9 mediated IFN-I production was required for the therapeutic activity of multiple chemotherapeutic drugs (269). To activate potent anti-tumor immune responses, endogenous tumor-derived NAs have to escape degradation by nucleases and processing by NA editing enzymes. Upregulation of TREX1 following lethal irradiation of tumor cells was recently reported to prevent cGAS-STING mediated IFN-I production in tumor cells and tumor infiltrating DCs. Sequential delivery of sub-lethal doses of irradiation prevented TREX1 upregulation in tumor cells and allowed the activation of anti-tumor immune responses in an IFN-I-dependent manner, indicating that TREX1 may act as a rheostat to control radiation-induced tumor-derived immunostimulatory DNA (270). Multiple studies have also described the function of ADAR1 in the regulation of anti-tumor immunity. Deletion of ADAR1 in tumor cells delayed tumor growth and increased the therapeutic potential of immune checkpoint blockade therapies. ADAR1 was shown to edit RNA originating from endogenous retroelements in tumor cells and thus inhibited their ability to stimulate MDA5-mediated IFN-I production that is required for the activation of anti-tumor immune responses (271). Furthermore, epigenetic therapies that increase the expression of endogenous retroelements were potent stimulators of anti-tumor immune responses only when ADAR1 was depleted from cancer cells (272). The endolysosomal DNASE2A was also shown to negatively regulate the production of inflammatory cytokines induced upon the uptake of dying tumor cells by macrophages (273). Therefore, in the context of cancer, blocking cell-intrinsic nucleases and NA-editing enzymes that limit the immunostimulatory potential of endogenous NA may be of relevance to enhance anti-tumor immune responses. Alternately, homozygous deletion of *RNASEH2B* has been shown to occur in chronic lymphocytic leukemia (CLL) and other malignancies (274), Similarly, pathogenic mutations in SAMHD1 have been reported in up to 11% of CLL patients (275). These studies suggest tumor suppressor roles for SAMHD1 and RNase H2 and are currently under investigation.

Patients with cancer present elevated levels of circulatory cfDNA, mostly originating from tumors. Such elevation of cancer cfDNA arises from a combination of increased cancer cell abundance and the reduced activity of extracellular DNases in cancer patients' sera (276). In agreement with their overall low DNase activity, patients with cancer also showed increased levels of circulatory NETs (277, 278). The accumulation of NETs in mice and individuals with cancer contributed to metastasis by trapping circulatory cancer cells in target organs. Importantly targeting NETs by the administration of recombinant DNASE1 significantly inhibited metastasis in murine models of cancer (279, 280). DNASE1L3, an important regulator of several sources of cfDNA, was shown to be downregulated in multiple cancers. Importantly, a recent study that stratified patients with hepatocellular carcinoma found a significant correlation between reduced DNASE1L3 expression and poor survival (281). These results suggest that extracellular DNases are impaired in cancer patients and may contribute to tumor growth and metastasis.

## Conclusion and Therapeutic Avenues

Overall, comprehensive studies performed in patients and validated in experimental mouse models certify the prominent role of nucleases and NA-editing enzymes in the prevention of autoimmunity, autoinflammation, and malignancy. Not surprisingly, all of these pathogenic conditions involve irregularities in inflammation because NA degrading and processing enzymes function as sentinels to restrict the activation of PRRs, which are central to all inflammatory pathways. Prudently, a significant amount of research and pharmaceutical effort focuses on finding therapeutics to block PRR-mediated inflammatory pathways in autoimmune inflammatory conditions. However, the greatest challenge in targeting the PRR-pathway of autoinflammation is the unwanted subversion of immune-responses against opportunistic infections. Therefore, to achieve the best therapeutic outcomes, a clear understanding of the genetic aberrations that cause autoinflammatory conditions is essential, and yet, this path also has considerable challenges. For example, it is evident that monogenic aberrations in one or more intracellular nucleases and NA editing enzymes lead to interferonopathies mediated by the activation of the cytosolic cGAS-STING pathway, which makes these attractive targets for therapeutic intervention in such patients. However, caution needs to be exercised when using STING inhibitors in AGS patients because, as detailed in the previous section, disruption of cGAS and STING suppresses spontaneous anti-tumor immune surveillance (266). Indeed, several STING agonists are being tested as therapeutics in clinical trials for solid tumors (clinicaltrials.gov). Nonetheless, PRRs are key targets for therapeutic interventions. In fact, as a proof of principle, well-known antimalarial drugs—chloroquine and hydroxychloroquine (Plaquenil), are TLR-signaling inhibitors, successfully in clinical use as first line treatments for SLE, rheumatoid arthritis and Sjogren's syndrome (282). Not surprisingly, several small molecule inhibitors, antibodies, oligonucleotides, lipid-A analogs and microRNAs that interfere with TLR signaling are emerging as promising therapeutics for inflammatory autoimmune diseases, reviewed in detail by Gao et al. (283). Other important rational targets of therapeutic intervention include effector cytokines and their signaling components. Indeed, monoclonal antibodies against IFN-I (Sifalimumab) and IFNAR1 (Anifrolumab), and inhibitors of Janus Kinases (Baricitinib, Tofacitinib), have shown promising results in clinical trials for SLE and type-I interferonopathies (284–286).

Another notable methodology to tackle NA-mediated inflammation is to target the immunogenic NAs themselves by: (1) preventing immunogenic NA generation, or (2) promoting immunogenic NA degradation. As noted previously, retroelements constitute about 40% of the human genome, which are reverse transcribed within the cells and potentially generate highly immunogenic NAs due to their microbial origins. The immunogenic potential of these self-NAs is restricted by TREX1 and ADAR1, which play a central role in endogenous retroelement metabolism. Indeed, restriction of reverse transcriptase activity by using inhibitors (RTIs) like abacavir, lamivudine and zidovudine have shown clinical efficacy in reducing inflammatory responses in AGS patients (286). Extracellular nucleases, specifically, DNASE1L3 has emerged as a novel immunosuppressive cell-extrinsic agent that regulates extracellular immunogenic DNA (45, 182). Unlike intracellular nucleases that could be difficult to manipulate, exogenous supplementation of recombinant DNASE1L3 protein offers a viable therapeutic modality to prevent immunogenic DNA-dependent TLR signaling. This process would perhaps involve engineering and modifying DNASE1L3 protein to enhance its nuclease activity and increase its half-life in circulation.

In conclusion, clinical and experimental studies support the fact that regulation of NA-metabolism is at the heart of maintaining self-tolerance. Undoubtedly, nucleases and NA-editing enzymes have emerged as crucial sentinels in preventing autoinflammation and should be explored as viable therapeutic targets for autoimmune and inflammatory disease conditions.

## Author Contributions

PS wrote the section on extracellular nucleases. AG wrote the section on intracellular DNases. LS wrote the section on endogenous nucleic acid forms and sources. CS wrote the part on nucleic acids sensors and RNases and VS wrote the rest of the manuscript and together with CS compiled the entire manuscript. AF, PS, AG, and VS designed figures and the table and PB edited the manuscript and provided valuable advice throughout the writing process. All authors listed have made a substantial, direct, intellectual contribution to the work, and approved it for publication.

## Conflict of Interest

The authors declare that the research was conducted in the absence of any commercial or financial relationships that could be construed as a potential conflict of interest.
