# Corrigendum: The Role of Nucleases and Nucleic Acid Editing Enzymes in the Regulation of Self-Nucleic Acid Sensing

**DOI:** 10.3389/fimmu.2021.690853

**Published:** 2021-04-22

**Authors:** Pauline Santa, Anne Garreau, Lee Serpas, Amandine Ferriere, Patrick Blanco, Chetna Soni, Vanja Sisirak

**Affiliations:** ^1^ CNRS-UMR 5164, ImmunoConcEpT, Bordeaux University, Bordeaux, France; ^2^ Department of Pathology, New York University Grossman School of Medicine, New York, NY, United States; ^3^ Immunology and Immunogenetic Department, Bordeaux University Hospital, Bordeaux, France

**Keywords:** DNases, RNases, systemic lupus erythematosus, DNA sensing, RNA sensing, interferonopathies, aicardi goutieres syndrome, toll-like receptors

In the original article, there was a mistake in the legend for **Figure 2** as published. The correct legend for **Figure 2** was mistakenly omitted and replaced with the legend of figure 3. The correct legend appears below.

**Figure 2:**
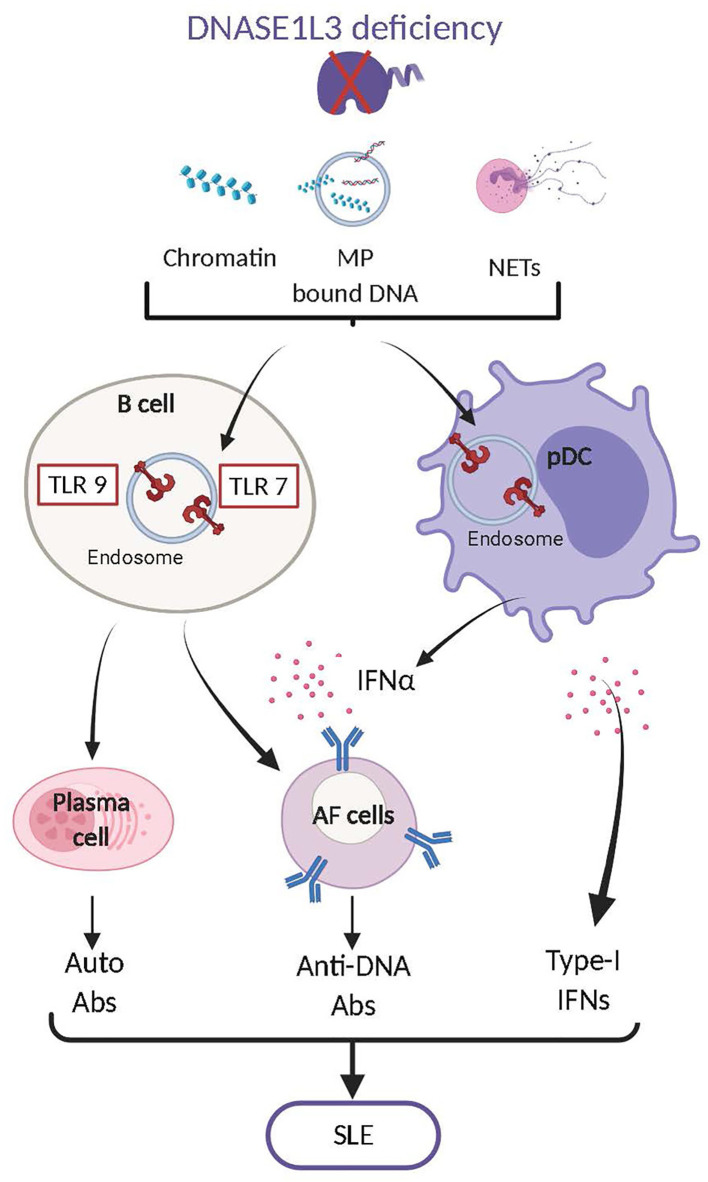
DNASE1L3 deficiency leads to systemic lupus erythematosus (SLE). DNASE1L3 deficiency leads to the accumulation of numerous forms of DNA including chromatin, MP associated DNA and NET-associated DNA. Accumulation of such DNA contributes to the aberrant activation of TLR7,9 in B cells and plasmacytoid dendritic cells (pDCs). In B cells TLR7,9 activation leads to their differentiation into plasma cells and antibody forming cells (AFC) that produce autoreactive antibodies mostly directed against dsDNA. In pDCs TLR7,9 activation induces the production of type I interferons (IFN-I) which also play an important role in the transition of B cells into AFC. The production of anti-dsDNA antibodies and of IFN-I will ultimately cause the development of Systemic Lupus Erythematosus (SLE).

DNASE1L3 deficiency leads to the accumulation of numerous forms of DNA including chromatin, MP associated DNA and NET-associated DNA. Accumulation of such DNA contributes to the aberrant activation of TLR7,9 in B cells and plasmacytoid dendritic cells (pDCs). In B cells TLR7,9 activation leads to their differentiation into plasma cells and antibody forming cells (AFC) that produce autoreactive antibodies mostly directed against dsDNA. In pDCs TLR7,9 activation induces the production of type I interferons (IFN-I) which also play an important role in the transition of B cells into AFC. The production of anti-dsDNA antibodies and of IFN-I will ultimately cause the development of Systemic Lupus Erythematosus (SLE).

The authors apologize for this error and state that this does not change the scientific conclusions of the article in any way. The original article has been updated.

